# “Two-stage resection of synchronous liver metastases in colorectal cancer leads to a survival benefit: a retrospective comparative cohort study”

**DOI:** 10.1007/s00423-025-03840-3

**Published:** 2025-08-28

**Authors:** Sebastian Lünse, Anne von Ruesten, Constanze Schneider, Stephan Gretschel, Andreas Loew, René Mantke

**Affiliations:** 1 Department of General and Visceral Surgery, University Hospital Brandenburg, Brandenburg Medical School Theodor Fontane, Hochstrasse 29, 14770 Brandenburg/Havel, Germany; 2 Clinical-Epidemiological Cancer Registry Brandenburg Berlin, Dreifertstrasse 12, 03044 Cottbus, Germany; 3 Department of General, Visceral, Thoracic and Vascular Surgery, University Hospital Ruppin-Brandenburg, Brandenburg Medical School Theodor Fontane, Fehrbelliner Straße 38, Neuruppin, 16816 Germany; 4 Faculty of Health Sciences Brandenburg, Joint Faculty of the Brandenburg University of Technology Cottbus-Senftenberg, the Brandenburg Medical School Theodor Fontane and the University of Potsdam, 14469 Potsdam, Germany; 5 Medical Department B, Division of Hepatology, Gastroenterology, Oncology, Hematology, Palliative Care, Endocrinology and Diabetes, University Hospital Ruppin-Brandenburg, Brandenburg Medical School Theodor Fontane, Fehrbelliner Straße 38, 16816 Neuruppin, Germany

**Keywords:** Colorectal cancer liver metastases, Operation sequences, Retrospective cohort study, Survival analysis

## Abstract

**Purpose:**

Colorectal cancer is the third most common cancer worldwide, with 15–25% of patients presenting synchronous liver metastases (UICC stage IV). Surgical resection remains crucial, but the optimal sequence for managing synchronous metastases is debated. This study evaluates the impact of different surgical strategies on survival in colorectal cancer patients with liver-only metastases (CRLM) and identifies factors influencing mortality.

**Methods:**

This retrospective cohort study analyzed CRLM patients from German cancer registries in Brandenburg and Berlin from 2017 to 2022, grouped by surgical treatment sequence: simultaneous primary tumor resection (PTR) and liver resection, PTR before liver resection, or liver resection before PTR. Kaplan-Meier and Cox regression analyses evaluated overall survival (OS) and the impact of systemic therapy and patient characteristics.

**Results:**

Among 23,394 colorectal cancer patients, 209 met inclusion criteria. Simultaneous resection was performed in 45% (*N* = 93), PTR before liver resection in 43% (*N* = 90), and liver resection first in 12% (*N* = 26). PTR before liver resection showed the best 5-year OS (68% vs. 53% for simultaneous surgery; HR 0.44, 95% CI = 0.22–0.88, *p* = 0.020). Simultaneous resection had the highest 30-day mortality (6.5%, *N* = 6). Postoperative chemotherapy significantly improved 5-year OS (66% vs. 57% for no chemotherapy; HR 0.45, 95% CI = 0.22–0.95, *p* = 0.036). Excluding 30-day mortality, survival differences diminished.

**Conclusion:**

Primary tumor resection before liver resection appears to improve long-term survival in CRLM patients. Simultaneous resection should be carefully considered due to increased mortality, particularly in older patients with lower performance status undergoing major liver surgery. Postoperative chemotherapy enhances survival, emphasizing the need for tailored treatment strategies.

**Supplementary Information:**

The online version contains supplementary material available at 10.1007/s00423-025-03840-3.

## Introduction

Colorectal cancer (CRC) is the third most common cancer worldwide and was the second leading cause of cancer-related death in 2022 [1]. The liver is the most common site of metastasis and 15–25% of patients have synchronous liver metastases (UICC stage IV disease) at the time of diagnosis [2].

Synchronous liver metastases, defined as metastases detected simultaneously with the primary colorectal tumor, present a clinical challenge due to their impact on treatment planning and overall prognosis. Surgical resection remains the cornerstone of curative-intent treatment for both primary tumors and liver metastases, but the optimal surgical treatment sequence and systemic therapy for synchronous liver metastases remains a topic of ongoing debate [3–6]. Recent advancements in surgical techniques [7–9] (e.g., laparoscopic and robot-assisted liver resection) and systemic treatment [10, 11] (e.g., more effective chemotherapies and immunotherapy) have rapidly expanded the treatment options available to patients in the last decade.

In this study, we present findings from a large cohort of stage IV colorectal cancer patients from the German cancer registry for Brandenburg and Berlin. While registry data is observational in nature, it provides valuable insights that go beyond those obtained from randomized controlled trials, which can lack generalizability [12, 13]. By encompassing an entire region, such data includes all patients and treatment providers, offering a comprehensive “real-world” perspective [14–16].

Our objective was to use cancer registry data to evaluate the impact of different surgical sequences on survival in UICC stage IV colorectal cancer patients with liver-only metastases and to identify factors influencing mortality, including systemic therapy and patient characteristics.

## Materials and methods

### Selection of study population

All patients diagnosed with stage IV colorectal cancer according to UICC were identified from the cancer registry for Brandenburg and Berlin (both Germany) between 1 January 2017 and 31 December 2022. We included only colorectal cancer patients with synchronous liver-only metastases (CRLM) who underwent primary tumor resection (PTR) and complete simultaneous or two-stage resection of liver metastases. The inclusion criteria specified that patients had to provide documented evidence of a tumor-free status (global R0) after the completion of the respective surgical therapy. Although there is no generally accepted interval to distinguish synchronous from metachronous metastases, patients with liver metastases present at the time of diagnosis of the colorectal cancer as well as patients with incidental liver metastases detected during surgery were classified as having synchronous CRLM. Patients with other metastatic sites, without therapy, only with systemic therapy, only with primary tumor resection, only with primary resection and systemic therapy, other therapy as well as patients without documented tumor-free status (no R0 resection of liver metastases) were excluded. PTR was defined as the surgical removal of the primary tumor within 12 months after diagnosis, using procedures classified under OPS codes 5-455.*, 5-456.*, 5-484.*, and 5-485.*. The extent of liver resection was defined with OPS codes 5-501.* as a minor intervention and 5-502.* as a major intervention.

### Statistical analysis

Patient characteristics were compared between treatment groups using Kruskal Wallis test for age and Pearson chi-square test for categorical variables and groupwise comparison. The survival of treatment groups was analyzed using Kaplan-Meier survival curves and log-rank tests. The follow-up period began at the time of diagnosis and continued until either death or 31 December 2022, whichever occurred first. Hazard ratios (HR) were calculated with Cox regression adjusting for sex (female, male), age at diagnosis (in years), type of liver metastases surgery (minor, major intervention), radiotherapy (yes, no), ECOG status (0, 1, 2, 3, unknown), localization of the primary tumor (colon, rectosigmoid/rectum), sequence of surgery (simultaneous PTR and liver resection, PTR before liver resection, liver resection before PTR), type of systemic therapy (none, pre-, peri-, postoperative), vital status (alive, died), cause of death (alive, tumor/non-tumor related, unknown), recurrence (no, yes), T-category (T1-4, TX), and N-category (N0-N3, NX). *P* values less than 0.05 were considered statistically significant. Analyses were performed with SPSS (version 24, IBM) and R (the R Foundation, version 4.3.0).

## Results

### Patient characteristics

**Fig. 1:**
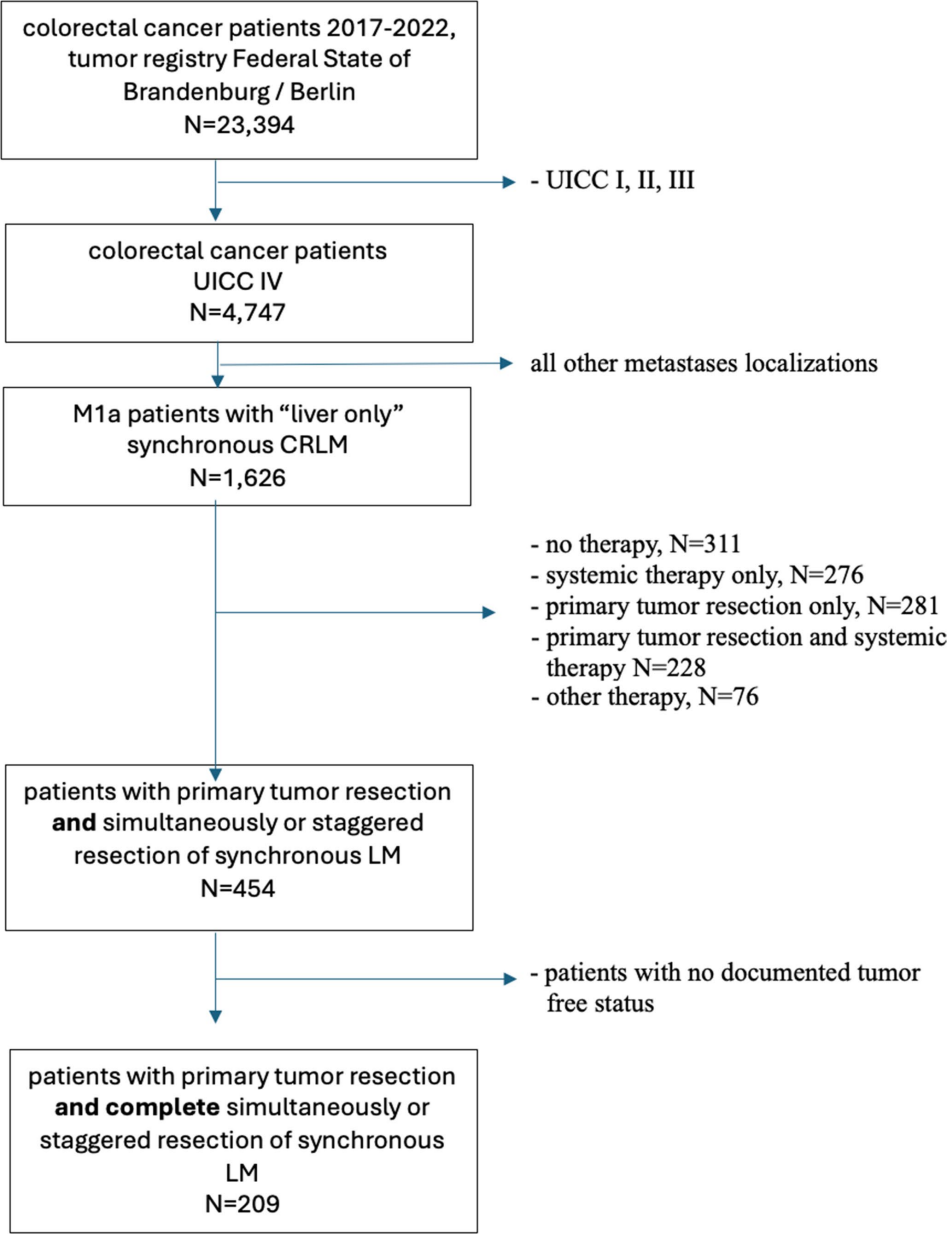
Flow chart illustrating selection of study population

A total of 23,394 patients with colorectal adenocarcinoma were identified from the cancer registry for the German federal states of Brandenburg and Berlin between 1 January 2017 and 31 December 2022. Of those, 4,747 patients were diagnosed with UICC stage IV. From this population, we identified 1,626 patients with liver-only synchronous CRLM (M1a disease), representing a total of 7% of all analyzed patients (Supplementary Fig. 1). Of those, 454 patients met the inclusion criteria regarding therapy. After excluding patients who had unknown or not documented tumor-free status, the number of patients included in our final analysis was 209 (Fig. 1). Of the 209 patients included in the final analysis, 93 patients (45%) underwent simultaneous PTR and liver resection, and 90 patients (43%) underwent PTR before liver resection, while 26 patients (12%) underwent liver resection before PTR (Table 1). The median age of the patients included in this study was 67 years (range 28–92 years). Slightly more than two-thirds of the patients analyzed were male (*n* = 144, 68.9%). The median age of the patients who received simultaneous PTR and liver resection was highest at 70 years, while patients who received liver resection before PTR had the lowest median age at 62.3 years (*p* = 0.03). Among all three surgical treatment groups analyzed, the majority of patients had an Eastern Cooperative Oncology Group (ECOG) performance status of 0 (*n* = 87, 41.6%), followed by ECOG status of 1 (*n* = 49, 23.4%).

**Table 1:** Characteristics of patients, tumor and treatment of the study population consisting of stage IV patients with liver-only synchronous CRLM and tumor-free status (global R0) after the respective surgical therapy (*N* = 209)

	Simultaneous PTR and liver resection (*N* = 93) 45%	PTR before liver (*N* = 90) 43%	Liver before PTR (*N* = 26) 12%	Overall (*N* = 209)	*p*-value*
Age (years)					
Mean (SD)	68.0 (11.4)	65.0 (11.5)	62.0 (10.4)	65.9 (11.5)	**0.030**
Median [Min, Max]	70.0 [38.0, 92.7]	66.3 [28.1, 84.8]	62.3 [41.7, 80.6]	67.1 [28.1, 92.7]	
Sex					
Females	33 (35.5%)	26 (28.9%)	6 (23.1%)	65 (31.1%)	0.402
Males	60 (64.5%)	64 (71.1%)	20 (76.9%)	144 (68.9%)	
State of residence					
Berlin	40 (43.0%)	50 (55.6%)	12 (46.2%)	102 (48.8%)	0.227
Brandenburg	53 (57.0%)	40 (44.4%)	14 (53.8%)	107 (51.2%)	
ECOG					
ECOG 0	37 (39.8%)	36 (40.0%)	14 (53.8%)	87 (41.6%)	0.341
ECOG 1	24 (25.8%)	22 (24.4%)	3 (11.5%)	49 (23.4%)	
ECOG 2	1 (1.1%)	7 (7.8%)	1 (3.8%)	9 (4.3%)	
ECOG 3	3 (3.2%)	1 (1.1%)	1 (3.8%)	5 (2.4%)	
ECOG unknown	28 (30.1%)	24 (26.7%)	7 (26.9%)	59 (28.2%)	
Type of liver metastases surgery					
Minor intervention	51 (54.8%)	25 (27.8%)	8 (30.8%)	84 (40.2%)	**< 0.001**
Major intervention	42 (45.2%)	65 (72.2%)	18 (69.2%)	125 (59.8%)	
Systemic therapy					
None	36 (38.7%)	22 (24.4%)	5 (19.2%)	63 (30.1%)	**< 0.001**
Peri-OP	5 (5.4%)	10 (11.1%)	4 (15.4%)	19 (9.1%)	
Post-OP	42 (45.2%)	19 (21.1%)	6 (23.1%)	67 (32.1%)	
Pre-OP	10 (10.8%)	39 (43.3%)	11 (42.3%)	60 (28.7%)	
Radiotherapy					
No	70 (75.3%)	59 (65.6%)	7 (26.9%)	136 (65.1%)	**< 0.001**
Yes	23 (24.7%)	31 (34.4%)	19 (73.1%)	73 (34.9%)	
Localization					
Colon	65 (69.9%)	57 (63.3%)	5 (19.2%)	127 (60.8%)	**< 0.001**
Rectosigmoid/Rectum	28 (30.1%)	33 (36.7%)	21 (80.8%)	82 (39.2%)	
Postoperative mortality within 30 days					
No	87 (93.5%)	90 (100%)	26 (100%)	203 (97.1%)	**0.021**
Yes	6 (6.5%)	0 (0%)	0 (0%)	6 (2.9%)	
Vital status ^1^					
Alive	64 (68.8%)	70 (77.8%)	20 (76.9%)	154 (73.7%)	0.358
Died	29 (31.2%)	20 (22.2%)	6 (23.1%)	55 (26.3%)	
Cause of death ^1^					
Alive	64 (68.8%)	70 (77.8%)	20 (76.9%)	154 (73.7%)	0.535
Tumor-related death	8 (8.6%)	6 (6.7%)	1 (3.8%)	15 (7.2%)	
Non-tumor-related death	4 (4.3%)	1 (1.1%)	2 (7.7%)	7 (3.3%)	
Unknown	17 (18.3%)	13 (14.4%)	3 (11.5%)	33 (15.8%)	
Recurrence ^1^					
No	68 (73.1%)	55 (61.1%)	21 (80.8%)	144 (68.9%)	0.081
Yes	25 (26.9%)	35 (38.9%)	5 (19.2%)	65 (31.1%)	
T-category					
T1	1 (1.1%)	1 (1.1%)	0 (0%)	2 (1.0%)	0.723
T2	8 (8.6%)	4 (4.4%)	0 (0%)	12 (5.7%)	
T3	57 (61.3%)	63 (70.0%)	18 (69.2%)	138 (66.0%)	
T4	22 (23.7%)	20 (22.2%)	7 (26.9%)	49 (23.4%)	
TX	5 (5.4%)	2 (2.2%)	1 (3.8%)	8 (3.8%)	
N-category					
N0	17 (18.3%)	20 (22.2%)	1 (3.8%)	38 (18.2%)	0.071
N1	33 (35.5%)	35 (38.9%)	8 (30.8%)	76 (36.4%)	
N2	36 (38.7%)	34 (37.8%)	13 (50.0%)	83 (39.7%)	
N3	1 (1.1%)	0 (0%)	0 (0%)	1 (0.5%)	
NX	6 (6.5%)	1 (1.1%)	4 (15.4%)	11 (5.3%)	

*ECOG* Eastern Cooperative Oncology Group, *OPS* operation and procedure code (according to German procedure classification), *SD* standard deviation. ECOG performance status: 0 = fully active, able to carry on all predisease performance without restriction; 1 = restricted in physically strenuous activity but ambulatory and able to carry out work of a light or sedentary nature; 2 = ambulatory and capable of all self-care but unable to carry out any work activities; 3 = capable of only limited self-care. *Kruskal Wallis Test (age), Pearson chi-square test for categorical variables; ^1^ Events considered up to the cut-off date: December, 31th, 2022

### Catmentharacteristics of tumor and Treatment

With regard to the extent of liver surgery, minor procedures (OPS 5-501) were performed significantly more frequently in the group of simultaneous PTR and liver resection than in the group of PTR before liver resection as well as in the group of liver resection before PTR. Major interventions in liver metastasis surgery (OPS 5-502) were performed significantly more frequently within the respective group in primary tumor resections before liver resection (*n* = 65, 72.2%) and in liver resections before PTR (*n* = 18, 69.2%). The analysis of the respective surgical intervention group with regard to systemic therapy showed that patients who underwent simultaneous PTR and liver resection received significantly less perioperative (*n* = 5, 5.4%) and preoperative chemotherapy (*n* = 10, 10.8%). In addition, the proportion of patients who did not receive any form of systemic therapy was highest in this group (*n* = 36, 38.7%). The chemotherapy treatment regimens were similar in the two groups of PTR before liver resection and liver resection before PTR. Among the 146 patients who received systemic therapy, 62% (*n* = 90) completed the initially planned regimen, while therapy was discontinued in 16% (*n* = 23). In 23% of cases (*n* = 33), the reason for therapy termination was unclear. The most frequently administered regimens were FOLFOX (*n* = 45, 31%), Capecitabine (*n* = 15, 10%), and FOLFOX plus Bevacizumab (*n* = 15, 10%). A wide variety of additional combinations were used at lower frequencies (Supplementary Table 1). Radiotherapy was performed significantly less frequently in the groups of simultaneous PTR and liver resection (*n* = 23, 24.7%) and PTR before liver resection (*n* = 31, 34.4%). Patients who underwent liver resection before PTR received significantly more frequently radiotherapy (*n* = 19, 73.1%). Considering the localization of the tumor, the colon was significantly more frequently affected in the groups of simultaneous PTR and liver resection (*n* = 65, 69.9%) as well as PTR before liver resection (*n* = 57, 63.3%). Liver resection before PTR was significantly more common in rectal or rectosigmoid tumors (*n* = 21, 80.8%). A significantly increased postoperative mortality within 30 days was observed in the group of patients who underwent PTR and liver resection simultaneously (*n* = 6, 6.5%), while no cases occurred in the two groups of PTR before liver resection and liver resection before PTR. At the cut-off date (31 December, 2022), 154 of 209 patients (73.7%) were alive, with slightly higher survival rates in the staged resection groups (*n* = 70, 77.8% for PTR before liver resection and *n* = 20, 76.9% for liver-first) compared to simultaneous resection (*n* = 64, 68.8%). Regarding cause of death, tumor-related mortality occurred in 15 patients (7.2%) overall, with slightly higher proportions in the simultaneous group (*n* = 8, 8.6%) compared to the sequential groups (*n* = 6, 6.7% for PTR before liver resection and *n* = 1, 3.8% for liver-first). Non-tumor-related deaths were observed in 7 patients (3.3%), and in 33 patients (15.8%) the cause of death remained unknown. Tumor recurrence was documented in 65 patients (31.1%) overall. The recurrence rates varied across treatment strategies: the highest rate of 38.9% (*n* = 35) was observed in the PTR before liver resection group, followed by 26.9% (*n* = 25) in the simultaneous group, and 19.2% (*n* = 5) in the liver-first group. The majority of tumors were staged as T3 (*n* = 138, 66.0%), and advanced nodal disease was frequent, with 39.7% (*n* = 83) classified as N2, followed by N1 in 36.4% (*n* = 76) of patients (Table 1).

### Effect of surgical treatment sequence on survival

**Fig. 2:**
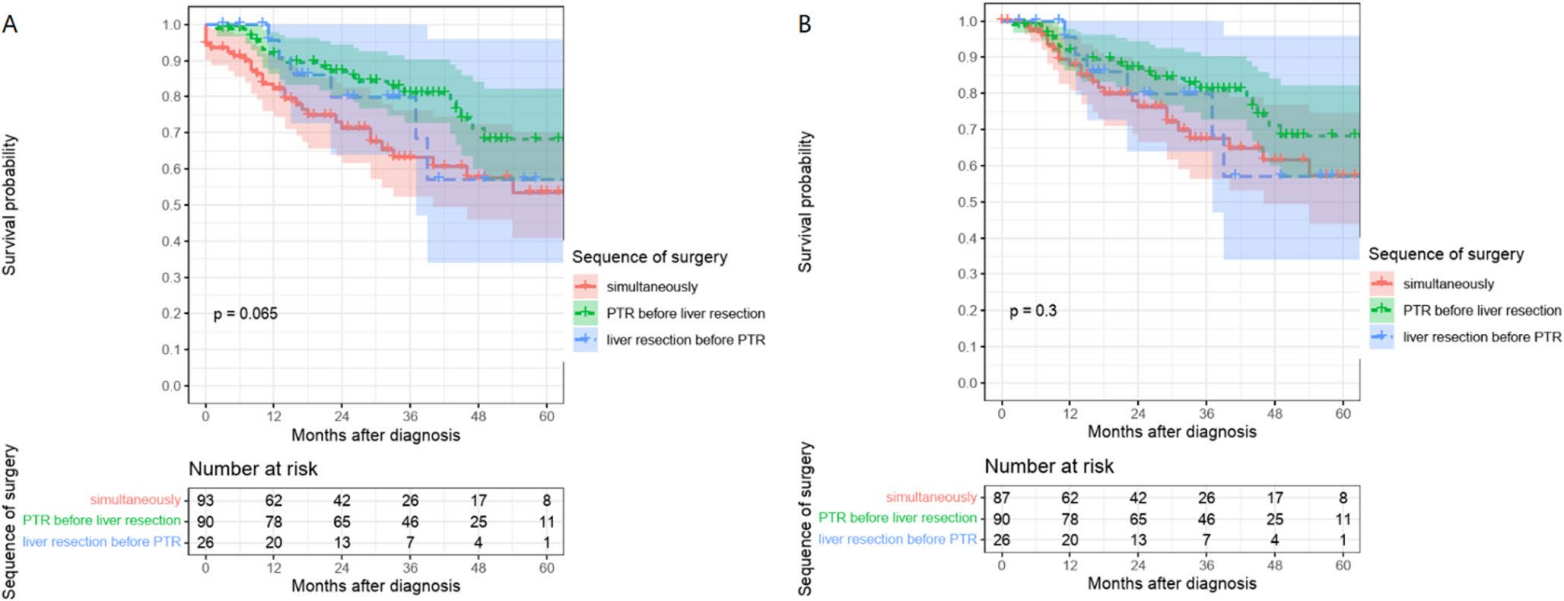
(**A**) Kaplan-Meier analysis illustrating significant overall survival differences between PTR before liver resection and simultaneous PTR and liver resection in UICC stage IV colorectal cancer patients with liver-only synchronous CRLM (*p*=0.022, *g*roupwise comparisonLog*-*Rank). (**B**) After excluding postoperative 30-day mortality, no significant differences in overall survival were observed between the surgical treatment sequences

Colorectal carcinoma patients with UICC stage IV and liver-only metastases who underwent primary tumor resection before liver resection had a significantly better overall survival compared to the simultaneous PTR and liver resection group (Fig. 2A). This was confirmed by cox regression analysis: HR univariable 0.51, 95% CI 0.29–0.91, *p* = 0.022 and HR multivariable 0.44, 95% CI 0.22–0.88, *p* = 0.020 (Table 2). The patient group of PTR before liver resection had the best 5-year overall survival (OS) of 68%, while patients with simultaneous PTR and liver resection had an OS of 53%. Patients who underwent liver resection before PTR had an OS of 57% (overall *p* = 0.065, Log-Rank). After excluding postoperative 30-day mortality, no significant differences in overall survival were found between the surgical treatment sequences (Fig. 2B). The exclusion of 30-day mortality resulted in an increase in 5-year overall survival in the simultaneous resection group from 53 to 57% (overall *p* = 0.3, Log-Rank). The analysis revealed that the only patients who died were those in the group that underwent simultaneous PTR and liver resection (*N* = 6). A subgroup analysis demonstrated that the patients in this group had a higher average age (77.4 years versus 69.0 years) and ECOG (0% versus 42.5% ECOG 0) status (Supplementary Table 2). The majority of liver resections in deceased patients were classified as major interventions (66.7% versus 43.7%). With regard to recurrence-free survival (RFS), the results of the cox regression analysis revealed no significant differences between the surgical treatment sequences (Supplementary Fig. 2). Regardless of this, the univariable analysis showed that the age of the patient was a significant predictor of mortality risk (HR univariable 1.03, 95% CI 1.00-1.05, *p* = 0.030).

**Table 2:** Univariate and multivariate analysis of the relationship between patient factors and different therapies on mortality in patients in UICC stage IV colorectal cancer with liver-only synchronous CRLM based on Cox regression. CI – confidence interval; HR – hazard ratio

Factor	Subgroup	*N* (%)	HR (univariate), 95% CI	HR (multivariate), 95% CI
Sequence of surgery	PTR and liver simultaneously	93 (44.5)	-	-
	PTR before liver	90 (43.1)	**0.51 (0.29–0.91**, *p* **= 0.022)**	**0.44 (0.22–0.88**, *p* **= 0.020)**
	Liver before PTR	26 (12.4)	0.69 (0.29–1.66, *p* = 0.404)	0.86 (0.30–2.44, *p* = 0.778)
Sex	Females	65 (31.1)	-	-
	Males	144 (68.9)	0.83 (0.48–1.45, *p* = 0.521)	0.84 (0.46–1.52, *p* = 0.563)
Age	Mean (SD)	65.9 (11.5)	**1.03 (1.00-1.05**, *p* **= 0.030)**	1.02 (0.99–1.05, *p* = 0.162)
Type of liver metastases surgery	Minor intervention (OPS 5-501)	84 (40.2)	-	-
	Major intervention (OPS 5-502)	125 (59.8)	0.97 (0.56–1.66, *p* = 0.910)	1.01 (0.55–1.88, *p* = 0.963)
Systemic therapy	None	63 (30.1)	-	-
	Peri-OP	19 (9.1)	0.58 (0.22–1.53, *p* = 0.271)	1.17 (0.35–3.97, *p* = 0.797)
	Post-OP	67 (32.1)	**0.40 (0.20–0.79**, *p* **= 0.009)**	**0.45 (0.21–0.95**, *p* **= 0.035)**
	Pre-OP	60 (28.7)	0.57 (0.30–1.10, *p* = 0.093)	1.00 (0.44–2.25, *p* = 0.999)
Radiotherapy	No	136 (65.1)	-	-
	Yes	73 (34.9)	0.82 (0.46–1.45, *p* = 0.490)	0.78 (0.40–1.52, *p* = 0.474)
ECOG	ECOG 0	87 (41.6)	-	-
	ECOG 1	49 (23.4)	1.83 (0.93–3.60, *p* = 0.078)	1.59 (0.74–3.40, *p* = 0.232)
	ECOG 2	9 (4.3)	1.18 (0.27–5.12, *p* = 0.821)	0.96 (0.21–4.33, *p* = 0.955)
	ECOG 3	5 (2.4)	2.12 (0.49–9.15, *p* = 0.315)	0.87 (0.16–4.69, *p* = 0.869)
	ECOG unknown	59 (28.2)	1.71 (0.88–3.33, *p* = 0.112)	1.44 (0.68–3.08, *p* = 0.341)
Localization	Colon	127 (60.8)	-	-
	Rectosigmoid/Rectum	82 (39.2)	0.82 (0.47–1.42, *p* = 0.475)	1.07 (0.56–2.06, *p* = 0.835)
T-category	T3	138 (66.0)	-	-
	< T3	14 (6.7)	0.82 (0.25–2.66, *p* = 0.739)	0.59 (0.17–2.02, *p* = 0.398)
	T4	49 (23.4)	1.23 (0.67–2.26, *p* = 0.499)	1.14 (0.60–2.18, *p* = 0.690)
	TX	8 (3.8)	1.34 (0.32–5.58, *p* = 0.688)	1.90 (0.30-11.93, *p* = 0.494)
N-category	N0	38 (18.2)	-	-
	N1	76 (36.4)	0.86 (0.42–1.75, *p* = 0.672)	0.92 (0.41–2.05, *p* = 0.834)
	≥N2	84 (40.2)	0.81 (0.40–1.66, *p* = 0.571)	0.92 (0.40–2.09, *p* = 0.834)
	NX	11 (5.3)	0.70 (0.16–3.12, *p* = 0.637)	0.35 (0.05–2.51, *p* = 0.296)

### Effect of systemic therapy on survival

**Fig. 3:**
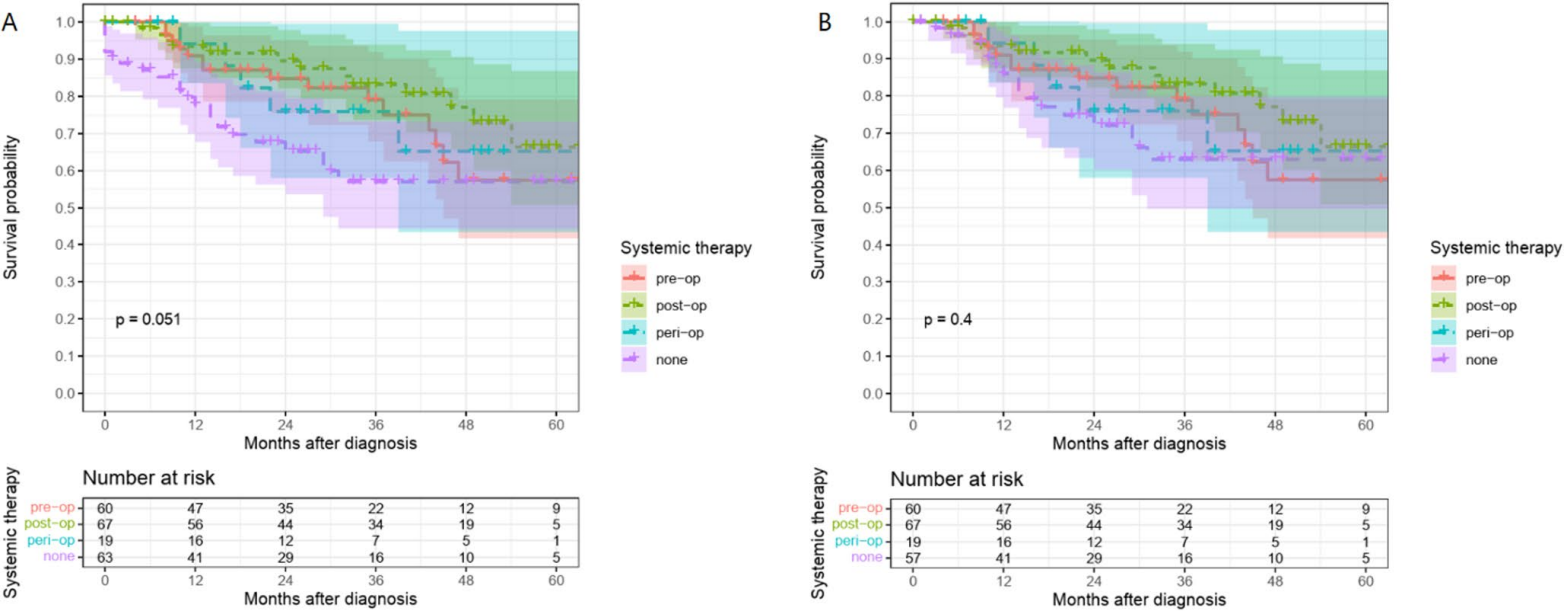
(**A**) Kaplan-Meier analysis illustrating significant overall survival differences between postoperative and non-systemic therapy (*p*=0.01,*g*roupwise comparisonLog*-*Rank). (**B**) After excluding postoperative 30-day mortality, no significant differences in overall survival with regard to systemic therapy were observed

With regard to systemic therapy in UICC stage IV cancer patients with liver-only metastases, the implementation of postoperative chemotherapy resulted in significantly better overall survival compared to patients who did not receive chemotherapy (Fig. 3A). This was confirmed by cox regression analysis: HR univariable 0.40, 95% CI 0.20–0.79, *p* = 0.009 and HR multivariable 0.45, 95% CI 0.21–0.93, *p* = 0.035 (Table 2). The patient group that received postoperative chemotherapy had the best 5-year OS of 66%, followed by the group that received perioperative chemotherapy at 65%. The overall survival rate of patients who received preoperative chemotherapy was 57%, which was equivalent to the survival rate of patients who did not receive chemotherapy (overall *p* = 0.051, Log-Rank). After excluding postoperative 30-day mortality, no significant differences in overall survival with regard to systemic therapy were detected (Fig. 3B). The exclusion of 30-day mortality resulted in an increase in 5-year overall survival in the none chemotherapy group from 57 to 63% (overall *p* = 0.4, Log-Rank).

## Discussion

The management of synchronous liver-only metastases in UICC stage IV colorectal cancer patients remains a complex, multidisciplinary and evolving field in oncological surgery. However, the optimal surgical approach and systemic therapy is still the subject of ongoing debate [3, 4, 17, 18]. This study provides valuable insights into the impact of surgical sequencing, patient characteristics, and chemotherapy on survival outcomes.

The analysis of our real-world data study among 209 stage IV colorectal cancer patients with synchronous liver-only metastases revealed that simultaneous PTR and liver resection (45% of cases) and PTR before liver resection (43%) are well-established surgical procedures for mainly colon cancer. The liver-first approach (12%) was predominantly employed in patients with rectal cancer who necessitated a major liver resection. This finding aligns with the conclusions of other retrospective studies, which demonstrated that patients who underwent liver-first resections had a higher hepatic tumor burden [18, 19]. Consequently, the liver-first approach should be the treatment of choice for patients with multiple metastases. However, it is important to note that this subgroup included only 26 patients in this study. The small sample size limits the statistical power and may increase the risk of random effects or selection bias. Thus, the observed survival benefit should be considered hypothesis-generating rather than conclusive, and validation in larger, prospective cohorts is warranted.

The results of the present study indicate that the conventional two-stage surgical approach with primary tumor resection before liver resection is associated with the best long-term outcome. The 5-year overall survival rate for these patients was 68%, which was significantly higher than in the group of patients who underwent simultaneous resection. In Contrast, a study by Brouquet *et* al. including 156 patients revealed a 5-year overall survival of 45% with no significant differences between the three surgical approaches [20]. Furthermore, a retrospective analysis by Vallance *et* al. among 1,830 patients in the English National Health Service (NHS) found no survival difference of PTR before liver resection compared to the liver-first or simultaneous strategy [21]. In addition, a pairwise network meta-analysis of 10,848 patients by Ghiasloo *et* al. showed no significant differences in 5-year OS between the three surgical approaches [18].

However, a striking finding emerged from our analysis: there was a significantly increased postoperative mortality within 30 days of 6.5% in patient who underwent simultaneous PTR and liver resection. The subgroup analysis demonstrated that patients with 30-day mortality were older and had a worse Eastern Cooperative Oncology Group (ECOG) performance status. Nevertheless, the implications of this study are limited by the fact that only 50% of cases in this subgroup had known ECOG status. Furthermore, a major liver resection was performed in two-thirds of the cases. A prospective multi-institutional study by Snyder *et* al. involving more than 30,000 patients confirmed an increased postoperative mortality rate after simultaneous resection [22]. Several previous studies have confirmed an elevated mortality rate in cases of simultaneous resection [20, 23, 24], while others have found no significant difference [25, 26]. Therefore, it is essential to emphasize that simultaneous resection should not be routinely recommended. This strategy should only be considered when the benefits clearly outweigh the cumulative risks of staged surgery, including those related to multiple anesthetic exposures, repeated hospitalizations, and delays in systemic therapy. In the absence of a compelling survival advantage, a staged approach remains the preferred option for many patients. In accordance with the results of previous studies, our findings suggest that careful patient selection is crucial for the optimal management of patients with liver-only metastases of stage IV colorectal carcinoma. An individually tailored surgical approach involving a hepatobiliary team is recommended to optimize outcomes. Based on the results of the present study, we propose specific criteria that may support the decision for a staged rather than simultaneous approach. These include advanced patient age, poor performance status (e.g., ECOG ≥ 1), the need for major liver resection (e.g., hemihepatectomy or bi-/trisectorectomy), or high hepatic tumor burden, such as multiple or bilobar colorectal liver metastases. In such scenarios, a staged approach may reduce perioperative risk and allow better physiological adaptation between surgical procedures. In addition, only minor liver resections should be performed in a simultaneous procedure.

As a secondary finding, the results of our study confirmed that postoperative chemotherapy significantly improved the 5-year overall survival rate compared to no chemotherapy (66% vs. 57%). This is consistent with the results of studies by Liang *et* al. including 6,533 patients and Sargent *et* al. among 20,898 patients, which both showed longer overall survival after adjuvant chemotherapy [27, 28]. Although our study results support existing evidence on the benefit of postoperative chemotherapy, they should be interpreted as exploratory due to the secondary nature of the analysis. Nevertheless, the optimal treatment regimens for perioperative chemotherapy remain the subject of ongoing research and controversial discussions. The possibility of treatment as part of an ongoing trial should therefore always be considered.

The present study offers a remarkable strength. The cancer registries encompass the German federal states of Brandenburg and Berlin, thereby ensuring a high level of completeness with regard to data on diagnoses, treatments, and oncological outcomes. Since 2016, the legal requirement for informed consent for case reporting has been suspended, with patients instead required to opt out of such reporting. It is estimated that the completeness of case reporting exceeds 90% [29].

Our analysis has several limitations. A selection bias results from the fact that the registry data do not characterize detailed information on comorbidities and differences in the primary tumor (e.g., obstruction, perforation, or bleeding) and the manifestation of liver metastases. Furthermore, this study provides no information regarding the indication generally adopted for the three different surgical approaches. This indication bias is difficult to eliminate completely, as it is often unknown or unobserved what constitutes an indication [30, 31]. By excluding cases without documented tumor-free status, a large proportion of patients (*N* = 245) were removed from our cohort. A major limitation is the fact that postoperative mortality within 30 days after the first surgical procedure was not recorded at all in the patient groups PTR before liver resection and liver resection before PTR. When comparing both postoperative mortality and long-term survival in this cohort, it should be noted that patients in both the liver-first and PFT groups must survive and recover sufficiently from the initial operation to complete treatment. Unavoidable, there will be patient ‘drop-outs’ from such complex and prolonged treatment pathways. Several studies have reported that between 16 and 35% of patients who undergo liver or primary tumor first surgery do not proceed to the second operation [32, 33]. The analysis of disease-specific survival was not undertaken due to the complexity and subjective nature of the cause of death, which is a combination of underlying and contributory causes. The majority of deaths are classified using ICD-10 C18-C20. Conversely, in our real-world data study, we accorded priority to the endpoint of overall survival, which was measured with both accuracy and objectivity.

## Conclusion

In conclusion, the present real-world data study suggests that the two-stage surgical approach, with primary tumor resection before liver resection, may be associated with improved long-term survival in UICC stage IV colorectal carcinoma patients with liver-only metastases. Patients should be carefully selected for simultaneous resection, as this procedure appears to be associated with increased postoperative mortality, particularly in older patients with lower performance status undergoing major liver surgery. Postoperative chemotherapy improves survival, underscoring the importance for an individually tailored treatment to optimize patient outcomes. However, future prospective studies with comprehensive oncological, clinical, and perioperative data are needed to determine the optimal surgical strategy for synchronous colorectal liver metastases.

## Supplementary Information

Below is the link to the electronic supplementary material.

Supplementary file1 (DOCX 216 KB)

## Data Availability

The study data will be available from the corresponding author for review upon a reasonable request.
