# Supplementary material for: “Two-stage resection of synchronous liver metastases in colorectal cancer leads to a survival benefit: a retrospective comparative cohort study”
Source: Langenbecks Arch Surg. 2025 Aug 28;410(1):253. doi: 10.1007/s00423-025-03840-3 (PMC12394268; doi:10.1007/s00423-025-03840-3)
Supplement: Supplementary file 1 — Supplementary file1 (DOCX 216 KB) [file 423_2025_3840_MOESM1_ESM.docx]

**Supplementary material**

**Supplementary Figure 1** Proportion of liver-only synchronous and other synchronous metastases in patients with colorectal cancer in the federal states of Brandenburg and Berlin (Germany) from 2017 to 2022 (*N*=23.394).

**Supplementary Figure 2** Kaplan-Meier analysis illustrating no significant differences in recurrence-free survival (RFS) between the surgical treatment sequences.

**Supplementary Table 1** Completion rate and chemotherapeutic agents in patients receiving systemic therapy (N=146).

**Supplementary Table 2** Comparison of deceased and surviving patients in the primary tumor resection and simultaneous resection of liver metastases group (*N*=93).


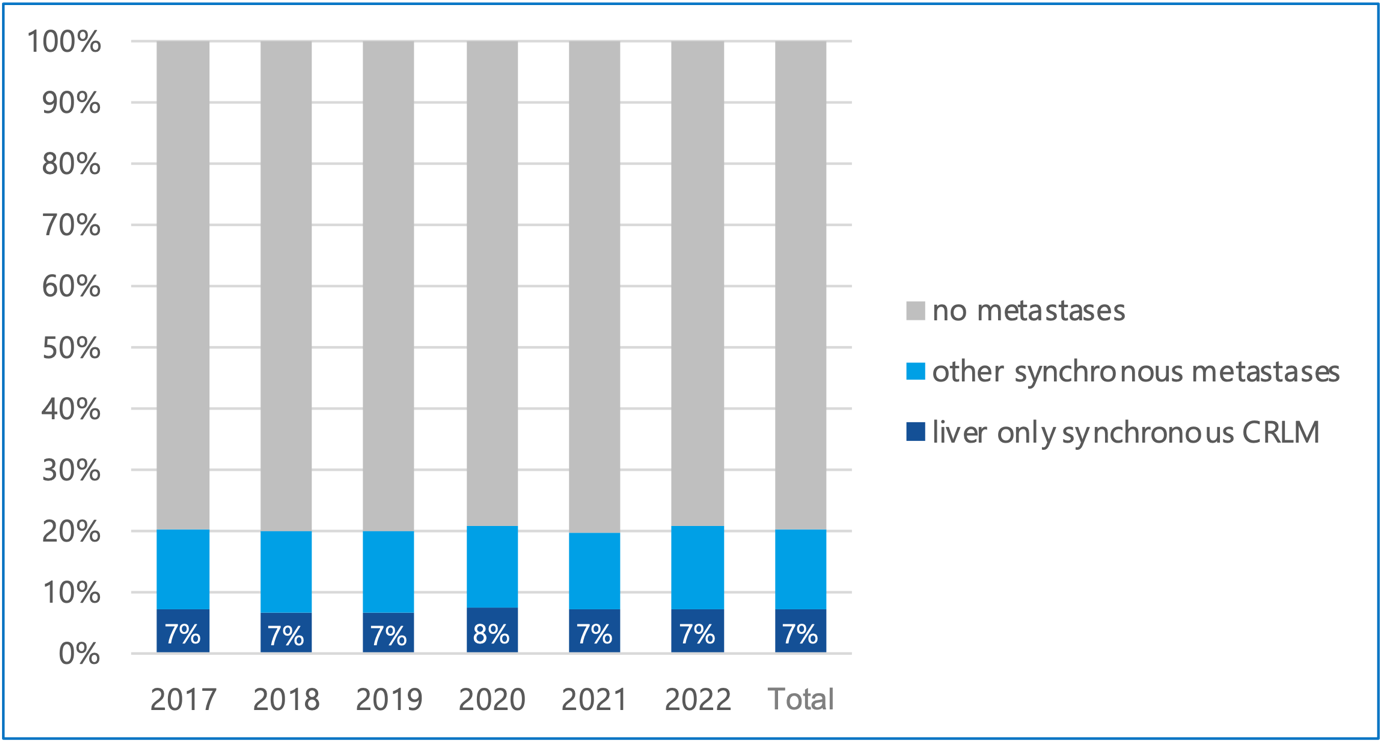


**Supplementary Figure 1** Proportion of liver-only synchronous and other synchronous metastases in patients with colorectal cancer in the federal states of Brandenburg and Berlin (Germany) from 2017 to 2022 (N=23.394).


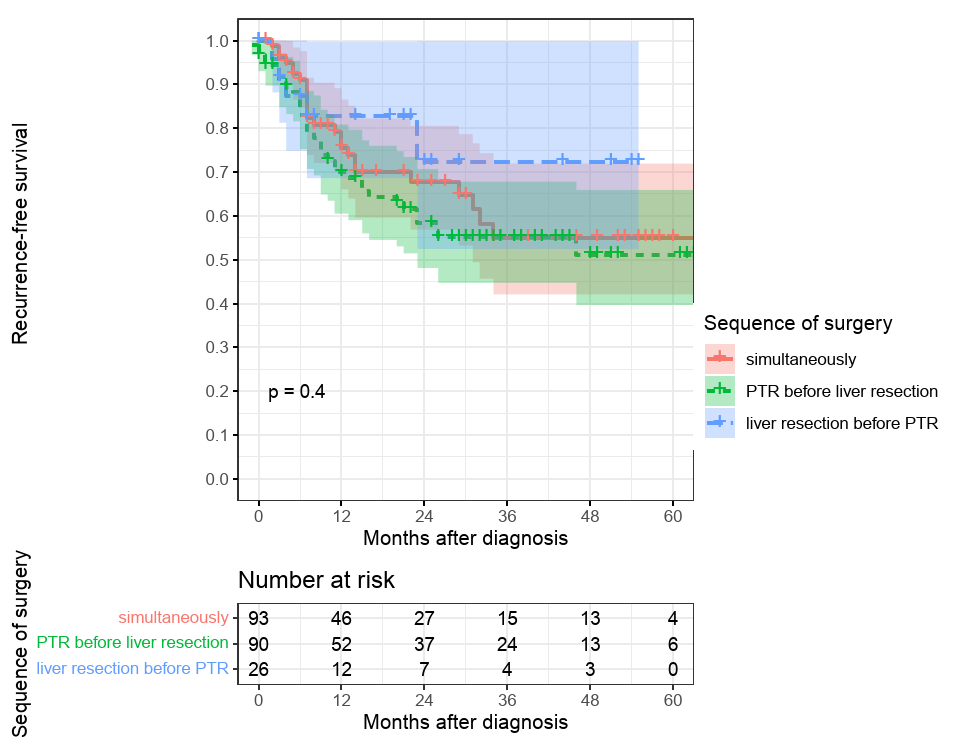


**Supplementary Figure 2** Kaplan-Meier analysis illustrating no significant differences in recurrence-free survival (RFS) between the surgical treatment sequences.

**Supplementary Table 1** Completion rate and chemotherapeutic agents in patients receiving systemic therapy (*N*=146).

|  | **N** | **%** |
| --- | --- | --- |
| **Completion rate^1^** |  |  |
| Therapy completion | 90 | 62% |
| Therapy cancellation | 23 | 16% |
| Reason for therapy termination unclear | 33 | 23% |
| **Agents (in descending order of frequency)^1^** |  |  |
| FOLFOX | 45 | 31% |
| Capecitabin | 15 | 10% |
| FOLFOX + Bevacizumab | 15 | 10% |
| FOLFIRI | 10 | 7% |
| FOLFIRI + Bevacizumab | 8 | 5% |
| Bevacizumab + FOLFOXFIRI | 8 | 5% |
| FOLFOX + Irinotecan | 7 | 5% |
| Xelox | 5 | 3% |
| FOLFOX + Cetuximab | 5 | 3% |
| 5-Fluorouracil | 4 | 3% |
| Bevacizumab | 3 | 2% |
| FOLFOX/FUFOX | 3 | 2% |
| FOLFOX-6 | 2 | 1% |
| FOLFIRI + Panitumumab | 2 | 1% |
| Rare combination | 2 | 1% |
| 5-Fluorouracil + Folin acid | 1 | 1% |
| 5-Fluorouracil + Folin acid + Bevacizumab | 1 | 1% |
| FOLFOX-4 | 1 | 1% |
| Folic acid + Oxaliplatin | 1 | 1% |
| Panitumumab | 1 | 1% |
| Pembrolizumab | 1 | 1% |
| Bevacizumab + Capecitabin | 1 | 1% |
| FOLFIRI + Cetuximab | 1 | 1% |
| Carboplatin + Etoposid | 1 | 1% |
| FOLFOX + Panitumumab | 1 | 1% |
| Not specified | 1 | 1% |

^1^Refers to first perioperative systemic therapy (subsequent changes or addition of agents are possible); FOLFOX: 5-Fluorouracil + Folinic acid + Oxaliplatin; FOLFIRI: 5-Fluorouracil + Folin acid + Irinotecan; FOLFOXFIRI: 5-Fluorouracil + Folin acid + Irinotecan + Oxaliplatin.

**Supplementary Table 2** Comparison of deceased and surviving patients in the primary tumor resection and simultaneous resection of liver metastases group (N=93).

|  | **Simultaneous PTR and liver resection, and postoperative mortality within 30 days after surgery (N=6)** | **Simultaneous PTR and liver resection, without postoperative mortality (N=87)** |
| --- | --- | --- |
| **Age (years)** |  |  |
| Median [Min, Max] | 77.4 [72.1, 84.1] | 69.0 [38.0, 92.7] |
| **ECOG** |  |  |
| ECOG 0 | 0 (0.0%) | 37 (42.5%) |
| ECOG 1 | 3 (50.0%) | 21 (24.1%) |
| ECOG 2 | 0 (0.0%) | 1 (1.1%) |
| ECOG 3 | 0 (0.0%) | 3 (3.4%) |
| ECOG unknown | 3 (50.0%) | 25 (28.7%) |
| **Type of liver metastases surgery** |  |  |
| Minor intervention (OPS 5-501) | 2 (33.3%) | 49 (56.3%) |
| Major intervention (OPS 5-502) | 4 (66.7%) | 38 (43.7%) |
